# Detection of Dinotefuran Residues in Fruits and Vegetables Using GC-MS/MS and Its Environmental Behavior and Dietary Risks

**DOI:** 10.3390/toxics13100816

**Published:** 2025-09-25

**Authors:** Chengling Ma, Jiamin Li, Peng Xue, Hao Zhang

**Affiliations:** 1 School of Public Health, Shandong Second Medical University, Weifang 250061, China; 2 Weifang Municipal Pollutant Emission Total Control Center, Weifang 250061, China

**Keywords:** neonicotinoid insecticides, residue monitoring, analytical method validation, food safety, ecotoxicology

## Abstract

This study developed a gas chromatography–tandem mass spectrometry (GC-MS/MS) method for detecting dinotefuran residues in fruits and vegetables. The modified extraction procedure employed solvent conversion for GC-MS/MS compatibility, achieving a linear range of 0.001–2.0 mg/kg (r^2^ > 0.999), a LOD of 0.003 mg/kg, and a LOQ of 0.01 mg/kg. Recovery rates ranged from 88.2% to 104.5% (RSD: 3.5–5.8%). The analysis of 18 commercial samples from Weifang, China, revealed the highest residues in nectarines (0.12 mg/kg) and lowest residues in cucumbers (0.02 mg/kg), with the dietary exposure risk assessment indicating hazard quotients well below safety thresholds. The literature review showed that dinotefuran has a shorter soil half-life (10–30 days) than most neonicotinoids, a low adsorption coefficient (Koc 30–50), high leaching potential, and significant toxicity to pollinators (LD_50_ = 0.023 μg/bee). The validated method provides reliable detection across diverse matrices, while the environmental behavior analysis highlights the need for the careful management of dinotefuran applications to minimize ecological impacts despite its favorable degradation profile compared to other neonicotinoids.

## 1. Introduction

Fruits and vegetables are vital components of a healthy diet, offering essential vitamins, trace elements, and phytochemicals that support physiological functions and reduce the risk of chronic diseases [1,2,3]. As public awareness of healthy eating grows, fruit and vegetable consumption has increased, raising concerns about their quality and safety. In modern agriculture, pesticides play a critical role in controlling pests and diseases, ensuring high yields and product quality. While the use of pesticides has significantly improved agricultural productivity and market supply, it has also led to growing concerns about pesticide residues in food [4,5]. Of particular concern are highly toxic or bioaccumulative insecticides, which can persist in produce, enter the human food chain, and pose potential health risks to consumers [6,7,8,9]. A 2023 monitoring study detected pesticide residues in 40–60% of fresh produce samples across many European countries, with 1–5% exceeding regulatory limits [10,11].

Dinotefuran, a third-generation neonicotinoid insecticide, is widely used in agriculture due to its broad-spectrum activity, high efficacy, and low dosage requirements. Its global market value reached USD 100–150 million in 2023, with usage increasing by 15% annually over the past five years, particularly in Asia-Pacific, North America, and Europe [12]. However, its potential health risks and environmental behavior have drawn significant attention from the scientific community. As a nicotinic acetylcholine receptor (nAChR) agonist, dinotefuran selectively targets the insect nervous system, disrupting neural transmission and causing paralysis and death in pests [13]. Compared to traditional pesticides, dinotefuran exhibits strong systemic activity, prolonged residual effectiveness, and high selectivity for target pests, making it particularly effective against piercing and sucking pests such as aphids, whiteflies, and leafhoppers. As a result, it is extensively applied in the cultivation of vegetables, fruits, tea, and other crops [14]. Despite its advantages, dinotefuran can enter the food chain through surface residues and systemic absorption by plants, posing potential health risks to humans. Studies indicate that dinotefuran and its metabolites may exhibit neurotoxicity, reproductive toxicity, and endocrine-disrupting effects [15]. Prolonged low-dose exposure has also been linked to an increased risk of neurodegenerative diseases [16]. Furthermore, dinotefuran poses significant toxicity to non-target beneficial organisms, such as bees, raising concerns about its ecological impacts [17,18]. Monitoring data from multiple countries have revealed instances of dinotefuran residues in agricultural products exceeding permissible limits, underscoring the urgent need for its regulated and scientific use. However, research on the residue characteristics and environmental behavior of dinotefuran in diverse fruit and vegetable matrices remains limited [19,20].

With advancements in analytical technologies, pesticide residue detection has progressed from traditional methods such as thin-layer chromatography and ultraviolet spectrophotometry to high-sensitivity techniques like high-performance liquid chromatography (HPLC), gas chromatography–mass spectrometry (GC-MS), and liquid chromatography–tandem mass spectrometry (LC-MS/MS) [21,22,23]. Among these, liquid chromatography is commonly used for dinotefuran residue analysis due to its compatibility with polar compounds. However, it has notable drawbacks, including a complex sample preparation and high analytical costs [24]. In contrast, gas chromatography–mass spectrometry (GC-MS) offers significant advantages for multi-component pesticide residue analysis, such as high separation efficiency, exceptional detection sensitivity, and precise qualitative and quantitative capabilities. Despite its potential, existing GC-MS-based methods for dinotefuran detection are hindered by several challenges, including labor-intensive sample preparation, significant matrix interference across diverse substrates, and suboptimal detection limits [25,26]. These issues are particularly pronounced in fruit and vegetable matrices due to their diverse physicochemical properties. The lack of standardized, efficient detection methods has impeded the routine monitoring and risk assessment of dinotefuran residues, highlighting the need for improved analytical approaches [26].

Systematic research on the environmental behavior of dinotefuran remains limited, particularly regarding its degradation pathways under varying environmental conditions, the toxicity of its degradation products, and its potential ecological impacts [27,28]. Such studies are essential for scientifically evaluating the environmental safety of dinotefuran and establishing a robust foundation for developing effective usage guidelines and regulatory policies.

Building on this context, the present study aims to develop an efficient GC-MS-based method for detecting dinotefuran residues in multi-matrix fruits and vegetables while analyzing its environmental behavior through a systematic literature review. Key innovations include modifying the sample preparation process and introducing a solvent conversion step to adapt GC-MS technology for detecting highly polar dinotefuran residues, as well as developing an analytical method suitable for diverse fruit and vegetable matrices and validating its application to real-world samples. Additionally, this study systematically examines the degradation, migration, and ecotoxicological characteristics of dinotefuran in the environment, comparing these with other neonicotinoid pesticides to provide a scientific basis for its proper use. The specific objectives are to develop and validate a GC-MS method for detecting dinotefuran residues; assess residue levels in commercially available fruits and vegetables and evaluate associated dietary exposure risks; and compare dinotefuran’s degradation rates, migration characteristics, and ecotoxicity with other neonicotinoids to inform environmental risk assessment and management. This research delivers reliable technical support for accurate dinotefuran detection and a robust scientific foundation for assessing its impacts on food safety and environmental health, offering valuable insights for risk management and the promotion of sustainable agricultural practices to protect consumer health.

## 2. Materials and Methods

### 2.1. Experimental Materials

This study adhered to the annual Food Safety Risk Monitoring Plan and followed the principle of random sampling. In June 2023, six representative seasonal fruits and vegetables commonly consumed in Weifang City, Shandong Province, China, were randomly collected from farmers’ markets (*n* = 6), supermarkets (*n* = 5), convenience stores (*n* = 4), and roadside stalls (*n* = 3). Weifang City was selected as the sampling site due to its status as a major agricultural production area in eastern China with diverse fruit and vegetable cultivation and representative pesticide application practices. The selected produce included cucumbers, spinach, celery, grapes, nectarines, and watermelons, with three samples collected for each type, totaling 18 samples.

For each sampling location, approximately 1–2 kg of each commodity was collected to ensure representative sampling. Samples were transported to the laboratory in insulated containers at 4 °C and processed within 24 h of collection. This pilot-scale sampling approach was designed to provide preliminary data on local dinotefuran residue levels, with the understanding that the limited sample size restricts the generalizability of findings.

The standards and reagents used in this study include a dinotefuran standard (DIN, 98% purity) obtained from Alta Scientific Co., Ltd., Tianjin, China, methanol (mass spectrometry grade) supplied by Thermo Fisher Scientific Inc., Waltham, MA, USA, and n-hexane (chromatography grade) from Merck Chemicals Co., Ltd., Darmstadt, Germany. Additionally, purified water was provided by Hangzhou Wahaha Group Co., Ltd, Hangzhou, China.

The instruments and parameters used in this study include a Shimadzu GCMS-TQ8050NX gas chromatography–mass spectrometer (Shimadzu (China) Co., Ltd., Shanghai, China) equipped with a DB-5 MS capillary column (30 m × 0.25 mm, 0.25 μm). Sample preparation utilized a Sigma1 1222 high-speed refrigerated centrifuge (Sigma Centrifuge [Yangzhou] Co., Ltd., Yangzhou, China), a VORTEX-5 vortex shaker (QiLinBeiEr Instrument Manufacturing Co., Ltd., Nantong, China), and an NBI-24D nitrogen evaporator (Shanghai BenAng Scientific Instrument Co., Ltd., Shanghai, China). Additionally, ultrapure water was supplied by an ultrapure water system from Shanghai Lichen Instrument Technology Co., Ltd, Shanghai, China.

### 2.2. Experimental Design

#### 2.2.1. Sample Pretreatment Method

The sample pretreatment method began with weighing 10.0 ± 0.1 g of homogenized fruit or vegetable sample. Each sample was processed using a high-speed homogenizer (IKA T25, Wilmington, NC, USA) with a capacity of 50–100 mL to ensure thorough tissue disruption. We added 10 mL of methanol and ground in a mortar for 1.5 min until the sample became a uniform fruit pulp. The homogenate was transferred to a 50 mL centrifuge tube and centrifuged at 12,500 r/min for 5 min to collect the supernatant. Given the polarity of dinotefuran and the requirements for GC-MS analysis, the supernatant was processed using a nitrogen evaporator. It was evaporated to near dryness at 40 °C under a nitrogen stream, redissolved in 2 mL of n-hexane, and mixed thoroughly. The solution was then filtered through a 0.22 μm membrane filter and prepared for detection by GC-MS. All samples were processed within 48 h of collection, and extracts were analyzed within 24 h of preparation to minimize degradation.

#### 2.2.2. Instrument Conditions

The GC-MS/MS detection utilized a Shimadzu GCMS-TQ8050NX with the following conditions. The temperature program began by holding at 50 °C for 1 min, followed by an increase at 25 °C/min to 125 °C (no hold), then increased at 10 °C/min to 260 °C, where it was held for 3 min. The carrier gas was high-purity helium with a flow rate of 1.69 mL/min, and a splitless injection mode was used. The injector temperature was set to 250 °C. The ionization source was electron impact (EI) with an ionization voltage of 70 eV, and the ion source temperature was 230 °C.

Detection was conducted in multiple reaction monitoring (MRM) mode, with a solvent delay time of 1.5 min [29]. For dinotefuran, the following transitions were monitored: *m*/*z* 157.0 > 113.0 (quantifier, CE 15 eV), *m*/*z* 157.0 > 99.0 (qualifier 1, CE 12 eV), and *m*/*z* 157.0 > 127.0 (qualifier 2, CE 10 eV). The qualifier/quantifier ion ratios were 0.85 ± 0.15 and 0.65 ± 0.15, respectively, and were used for confirmation of dinotefuran identity. The dwell time was set at 100 ms for each transition.

### 2.3. Method Validation

Linearity and Sensitivity: Calibration curves were constructed using blank matrix-matched standard solutions with concentration gradients of 0, 0.005, 0.05, 0.2, 0.8, 1.6, and 3.2 mg/kg. The linear range and sensitivity of the method were evaluated based on the linear correlation coefficient (r^2^) of the calibration curves.

Accuracy and Precision: Blank samples from six matrices (grape, nectarine, watermelon, cucumber, spinach, and celery) were spiked at three concentration levels (0.01, 0.1, and 1.0 mg/kg). Each concentration level was analyzed in six replicates, and the average recovery and relative standard deviation (RSD) were calculated to assess the accuracy and precision of the method.

Matrix Effect: The influence of the matrix on detection signals, either suppression or enhancement, was evaluated by comparing the slope ratio of the solvent standard curve to the matrix-matched standard curve. The matrix effect (ME) was calculated using the formula (1):

ME = (Slope_matrix/Slope_solvent) × 100%(1)

The matrix effect (ME) was interpreted as follows: an ME value of less than 100% indicates matrix suppression, greater than 100% indicates matrix enhancement, and approximately 100% suggests a negligible matrix effect, as referenced in studies [30,31,32].

### 2.4. Risk Assessment

This study employed the hazard quotient (HQ) to assess the dietary exposure risk of dinotefuran residues, including acute (short-term) exposure (acute HQ) and chronic (long-term) exposure (chronic HQ) [33,34]. An HQ value of ≤100% indicates that the dietary exposure level does not surpass the threshold for adverse effects in humans, signifying a low risk. Conversely, an HQ value > 100% suggests that the dietary exposure level exceeds the threshold, indicating a significant risk that requires attention [35,36].

(2) $$aHQ=\frac{ESTI}{ARfD}\times 100\%$$

(3) $$ESTI=\frac{C_{max}\times m}{BW}$$

(4) $$cHQ=\frac{EDI}{ADI}\times 100\%$$

(5) $$EDI=\frac{C_{m}\times m}{BW}$$

where ESTI is the estimated short-term intake of the pesticide (mg/kg); ARfD is the acute reference dose (mg/kg); Cmax is the maximum residue level of pesticide C (mg/kg); Cm is the average residue level of the pesticide (mg/kg); m is the average daily consumption of fruits and vegetables (kg/d) by Chinese residents; and BW is the average body weight of the population (kg). In this study, the value of m was derived from the highest recommended daily intake of fruits and vegetables in the Dietary Guidelines for Chinese Residents (2022), which specifies 0.350 kg/d for fruits and 0.500 kg/d for vegetables. The value of BW was set at 60 kg. EDI is the estimated daily intake of the pesticide (mg/kg); ADI is the acceptable daily intake (mg/kg). The ARfD and ADI values were obtained from the WHO/FAO Joint Meeting on Pesticide Residues (JMPR) [37] and the national standard GB2763-2021: National Food Safety Standard—Maximum Residue Limits for Pesticides in Food.

### 2.5. Literature Review Methodology

To systematically analyze the environmental behavior of dinotefuran, this study reviewed relevant research published between 2010 and 2024 by searching domestic and international literature databases, including PubMed, Web of Science, CNKI, and Scopus. The search keywords included “dinotefuran,” “environmental behavior,” “degradation,” “mobility,” “toxicity,” and “neonicotinoids.” A total of 82 high-quality studies were selected, focusing on the degradation of dinotefuran in soil and aquatic environments, its migration and transformation properties, and its toxic effects on non-target organisms. Additionally, a systematic comparison was made with other widely used neonicotinoid pesticides, such as imidacloprid, thiamethoxam, and acetamiprid. All data were obtained from published scientific literature or authoritative databases, such as PPDB (Pesticide Properties DataBase), EFSA (European Food Safety Authority), and EPA ECOTOX (Environmental Protection Agency Ecotoxicology Knowledgebase).

## 3. Results and Analysis

### 3.1. Validation Results

**Figure 1:**
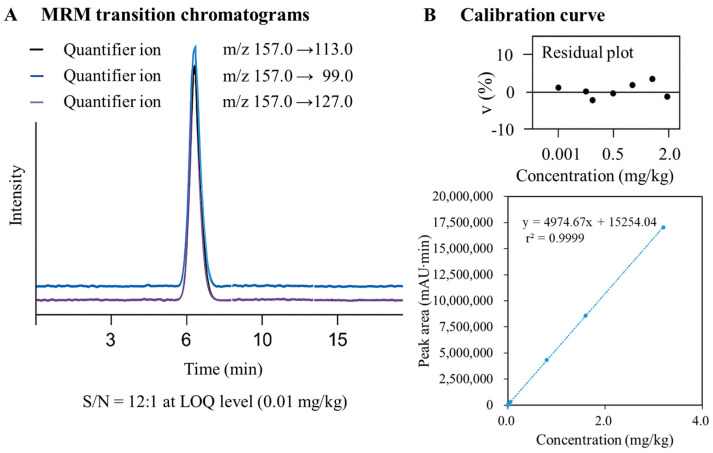
GC-MS/MS determination of dinotefuran: MRM chromatogram showing quantifier and qualifier transitions (**A**) and standard curve with residual plot (**B**).

As shown in Figure 1, the retention time of dinotefuran was approximately 6.14 min, with MRM transitions showing clear peaks for quantifier (*m*/*z* 157.0 > 113.0) and qualifier ions (*m*/*z* 157.0 > 99.0 and *m*/*z* 157.0 > 127.0). The signal-to-noise ratio at the LOQ level (0.01 mg/kg) was 12:1, exceeding the minimum requirement of 10:1. Dinotefuran demonstrated excellent linearity within the range of 0.001–2.0 mg/kg (calibration curve equation: y = 4974.67x + 15,254.04, r^2^ = 0.9999). The residual analysis showed a random distribution of residuals around zero with no systematic trend (residuals < ±5% across the linear range). The method’s limit of detection (LOD) and limit of quantification (LOQ) were determined to be 0.003 mg/kg and 0.01 mg/kg, respectively, complying with the EU guideline SANTE/11312/2021 requirements (LOQ ≤ 0.01 mg/kg) [38].

Table 1 presents the recovery and precision results at different concentration levels. For the three spiking levels (0.01, 0.1, and 1.0 mg/kg), the average recovery of dinotefuran across six matrices ranged from 88.2% to 104.5%, with relative standard deviations (RSDs, *n* = 6) between 3.5% and 5.8%. ANOVA results showed no significant differences in recovery rates among different matrices (F = 1.83, *p* = 0.12) or concentration levels (F = 2.04, *p* = 0.09). These results comply with internationally recognized standards (recovery: 70–120%; RSD ≤ 15%) [39,40]. The minimal variation in recovery rates among matrices demonstrates that the developed method is highly applicable to a variety of fruit and vegetable matrices [29].

The matrix effect (ME) values for the six fruits and vegetables analyzed in this study ranged from 94% to 105%. For cucumber, the ME was 105% (slight enhancement effect), for spinach it was 97% (mild suppression effect), for celery it was 94% (mild suppression effect), for grape it was 98% (negligible effect), for nectarine it was 101% (negligible effect), and for watermelon it was 102% (slight enhancement effect). These values meet the conventional standard requirements (80–120%) [30,31,32].

These findings emphasize the importance of using matrix-matched calibration standards to minimize matrix effects and their influence on detection results, ensuring the accuracy and reliability of the analytical method. The controlled matrix effects across diverse fruits and vegetables demonstrate the robustness of our sample preparation procedure.

As shown in Table 2, our GC-MS/MS method compares favorably with other published techniques for dinotefuran detection. While LC-MS/MS offers slightly better sensitivity (LOD 0.002 mg/kg), our method provides superior recovery rates (up to 104.5%) and is suitable for a wider range of matrices [41]. Compared to traditional GC-MS/MS approaches, our modified method achieves a 3-fold improvement in the LOD (0.003 vs. 0.01 mg/kg) and significantly better recovery rates (88.2–104.5% vs. 75.6–88.3%), demonstrating the effectiveness of our solvent conversion step for polar pesticide analysis [42,43].

### 3.2. Detection of Actual Samples

This study analyzed 18 market samples collected in 2023, categorized into three groups: leafy vegetables (6 samples, including spinach and celery), fruiting vegetables (3 samples, such as cucumber), and fruits (9 samples, including grape, nectarine, and watermelon) as detailed in Table 3. The samples included six varieties: cucumber, spinach, celery, grape, nectarine, and watermelon.

As shown in Table 3, nectarines exhibited the highest original pesticide residue level (0.12 ± 0.01 mg/kg), while cucumbers had the lowest (0.02 ± 0.002 mg/kg). Following spiking experiments (addition of 0.05 mg/kg), residue levels increased across all samples. For example, the pesticide residue level in cucumbers rose from 0.02 mg/kg to 0.09 mg/kg. The precision of the spiked measurements (RSD%: 2.4–9.3%) was better than that of the original data (RSD%: 8.6–9.6%), likely due to the higher concentration levels resulting in improved signal-to-noise ratios.

These findings indicate that nectarine and spinach samples had relatively high residue levels, suggesting potential residue risks that warrant attention. However, all detected residues were below the maximum residue limits (MRLs) established by Chinese national standards (GB 2763-2021, MRL for dinotefuran in fruits: 0.2–0.5 mg/kg; in vegetables: 0.1–0.3 mg/kg), EU regulations (MRL range: 0.01–0.1 mg/kg), and Codex Alimentarius standards (MRL range: 0.1–0.7 mg/kg) [39,40].

As shown in Table 4, our findings are generally consistent with both national and global monitoring data. The 100% detection rate in our study compared to much lower rates in larger monitoring programs (15–45%) reflects our small sample size and targeted sampling approach. The residue levels we detected fall within the ranges reported in broader monitoring studies, though our values for nectarines and leafy vegetables (spinach and celery) are in the upper percentiles of reported ranges, suggesting potentially higher usage patterns in the Weifang region.

Notably, while all our samples complied with Chinese and Codex MRLs, spinach and celery samples would exceed the more stringent EU MRLs (0.01 mg/kg), highlighting regional regulatory differences that could impact international trade.

### 3.3. Analysis of Environmental Degradation Characteristics

According to the literature review results, dinotefuran exhibits distinct environmental degradation characteristics [18,45]. As shown in Table 5, the half-life (DT50) of dinotefuran in soil ranges from 10 to 30 days, which is significantly shorter than that of imidacloprid (100–200 days) and thiamethoxam (30–100 days) and is comparable to acetamiprid (20–40 days). Key factors influencing its degradation rate include the temperature, humidity, pH, and microbial activity [46,47,48].

Studies indicate that dinotefuran degrades more rapidly under high temperatures (30–35 °C), high humidity (soil water content > 60%), and neutral to slightly alkaline conditions (pH 7–8) [48,49]. Microbial degradation is its primary degradation pathway in soil, with a half-life of 20–50 days. In contrast, photolysis (half-life of 4–14 days) and hydrolysis (half-life of approximately 360 days under neutral pH conditions) play a relatively minor role in its degradation under real-world conditions.

In aquatic environments, dinotefuran is highly stable, with a hydrolysis half-life exceeding 360 days under neutral pH conditions. However, it can rapidly degrade through photolysis under strong light exposure, with a half-life of approximately 4–14 days.

Within plants, dinotefuran primarily degrades through the metabolic enzyme system, with half-lives ranging from 2 to 12 days across different crops. This is notably shorter than the half-life of imidacloprid (7–25 days) and thiamethoxam (4–20 days).

### 3.4. Analysis of Environmental Migration Characteristics

According to the literature review results, dinotefuran exhibits distinct environmental degradation characteristics. As shown in Table 6, the soil adsorption coefficient (Koc) of dinotefuran ranges from 30 to 50, which is significantly lower than that of imidacloprid (200–300) and acetamiprid (150–200) and only slightly higher than that of thiamethoxam (60–100). This low adsorption coefficient indicates that dinotefuran has strong mobility and high leaching potential in soil. Consequently, it is easily transported into aquatic systems during rainfall or irrigation.

Dinotefuran’s high water solubility (39,830 mg/L), significantly greater than that of other neonicotinoid pesticides, further enhances its mobility in aquatic environments. Its groundwater ubiquity score (GUS) is 4.95, exceeding the threshold value (GUS > 2.8) that indicates leaching potential. The leaching risk of dinotefuran is substantially higher than that of imidacloprid (3.76) and acetamiprid (3.83) but slightly lower than that of thiamethoxam (5.18). These findings suggest that dinotefuran is highly prone to leaching in soil and may infiltrate groundwater through surface runoff and percolation, potentially contributing to water contamination.

Field studies have demonstrated that in regions with heavy rainfall or frequent irrigation, dinotefuran can rapidly penetrate into deep soil layers (50–100 cm) and potentially reach groundwater. In some sandy soil regions, the detection rate of dinotefuran in groundwater reaches 20–30%, significantly higher than that of imidacloprid (5–10%). These findings underscore the need for the cautious use of dinotefuran in water source protection zones and areas with high groundwater levels to mitigate the risk of water contamination.

### 3.5. Analysis of Ecotoxicological Characteristics

The literature review reveals that dinotefuran, as a neonicotinoid pesticide, exhibits distinct ecotoxicological characteristics (Table 7). Its acute contact toxicity to bees is LD_50_ = 0.023 μg/bee, which is relatively lower than that of imidacloprid (0.0039 μg/bee) and thiamethoxam (0.004 μg/bee) but significantly higher than that of acetamiprid (0.15 μg/bee). Although dinotefuran is considered one of the relatively less toxic neonicotinoids to bees, its potential risks cannot be overlooked, and the effects of subacute and chronic exposures require further research. Dinotefuran’s toxicity to aquatic invertebrates (LC_50_ = 0.1–1.0 mg/L) is relatively low, but high-concentration exposure could still pose risks to aquatic ecosystems. In comparison, imidacloprid and thiamethoxam exhibit greater aquatic toxicity, with thiamethoxam posing the highest threat to aquatic ecosystems (LC_50_ as low as 0.01–0.1 mg/L).

Literature studies indicate that the toxicity of dinotefuran to earthworms (EC_50_ = 5.2 mg/kg) is lower than that of thiamethoxam (2.8 mg/kg) but higher than that of imidacloprid (10.5 mg/kg) and acetamiprid (20.0 mg/kg). Metabolic studies reveal that dinotefuran primarily degrades in the environment into 1-methyl-3-(tetrahydro-3-furylmethyl)urea (UF) and demethylated dinotefuran (DN), both of which exhibit relatively low toxicity. In contrast, thiamethoxam metabolizes into clothianidin, another highly toxic neonicotinoid, thereby significantly increasing its ecological risks.

Overall, the acute toxicity of dinotefuran to bees is lower than that of imidacloprid and thiamethoxam. However, its toxicity to aquatic organisms, such as fish, is notably higher than that of acetamiprid. The potential impacts of dinotefuran on aquatic ecosystems, as well as the long-term effects of its metabolites, warrant further investigation.

### 3.6. Dietary Exposure Risk Assessment

This study evaluated the acute and chronic dietary exposure risks of dinotefuran based on the fruit and vegetable consumption data and pesticide residue analysis. The results (Table 8) show that the acute hazard quotients (aHQ: 0.07–0.39%) and chronic hazard quotients (cHQ: 0.07–0.36%) for all samples were far below the international safety threshold (HQ = 100%), indicating a minimal risk to human health from pesticide residues.

Among the samples, nectarines had the highest residue-related indicators (Cmax = 0.13 mg/kg, ESTI = 0.00078 mg/kg, aHQ = 0.39%). However, their maximum chronic intake (EDI = 0.00071 mg/kg) accounted for only 0.36% of the safety threshold. After the data adjustment, the pesticide residue risk ranking was as follows: nectarine > celery > spinach > watermelon > grape > cucumber. Overall, the risk is controllable, but enhanced monitoring of pesticide residues in nectarines is recommended.

Literature studies indicate that risk assessments should account for variations among different population groups. According to relevant studies, the hazard quotient (HQ) values for children (body weight 20 kg) are approximately three times higher than those for adults, while the chronic hazard quotient for vegetarian populations (average daily vegetable intake of 0.8 kg) is about 1.6 times higher than that of the general population [49,50]. This highlights that specific groups, such as children and vegetarians, may face elevated exposure risks and warrant particular consideration. Furthermore, the studies found that acute risk is inversely proportional to body weight, meaning individuals with specific dietary habits may face higher exposure risks. Consequently, assessments based on a daily intake of 0.350 kg of fruit and 0.500 kg of vegetables may underestimate acute dietary exposure risks for certain population groups [51].

## 4. Conclusions

This study successfully developed a sensitive, accurate, and reliable GC-MS/MS method for detecting dinotefuran residues in various vegetable and fruit matrices. The introduction of a solvent conversion step successfully overcame the technical challenges associated with analyzing polar pesticides, such as dinotefuran, using GC-MS/MS. The method validation demonstrated excellent performance characteristics, including a wide linear range (0.001–2.0 mg/kg), low detection limits (LOD: 0.003 mg/kg; LOQ: 0.01 mg/kg), and high recovery rates (88.2–104.5%).

The residue analysis of commercially available fruits and vegetables from Weifang, China, revealed generally low dinotefuran levels (0.02–0.12 mg/kg), with all samples below applicable MRLs. The dietary risk assessment indicated minimal health concerns, with hazard quotients below 0.4% of safety thresholds. However, the small sample size (*n* = 18) limits the generalizability of these findings, and particular attention should be given to potentially higher residue levels in nectarines and leafy vegetables.

The literature review highlighted dinotefuran’s distinctive environmental profile compared to other neonicotinoids: faster soil degradation (DT50: 10–30 days), higher water solubility (39,830 mg/L), lower soil adsorption (Koc: 30–50), and greater leaching potential (GUS: 4.95). While dinotefuran poses less risk to pollinators than imidacloprid or thiamethoxam, its significant mobility in soil and water warrants careful management, particularly in environmentally sensitive areas.

This research provides technical support for dinotefuran residue monitoring in agricultural products and a scientific foundation for its safe use and risk management. Future studies should focus on multi-residue methods for neonicotinoids, investigating transformation products under varying environmental conditions, and developing safer application strategies that balance agricultural productivity with environmental protection.

## Figures and Tables

**Table 1:** Recovery rates and precision of dinotefuran in six different matrices at various concentration levels.

Matrices	Addition Concentration (mg/kg)	Recovery Rate (%)	RSD (%, *n* = 6)
Grape	0.01	92.8	5.6
	0.10	96.5	4.3
	1.00	98.3	3.8
Nectarine	0.01	90.5	5.8
	0.10	94.7	4.5
	1.00	97.2	3.9
Watermelon	0.01	93.2	5.3
	0.10	97.8	4.2
	1.00	99.1	3.6
Cucumber	0.01	88.2	5.5
	0.10	91.3	4.7
	1.00	95.6	4.0
Spinach	0.01	95.3	5.7
	0.10	99.6	4.4
	1.00	105	3.5
Celery	0.01	91.5	5.2
	0.10	94.8	4.1
	1.00	97.4	3.7

**Table 2:** Comparison of different methods for dinotefuran residue detection.

Methods	Matrix Type	LOD (mg/kg)	LOQ (mg/kg)	Recovery Rate (%)	Reference
GC-MS	A variety of fruits and vegetables	0.003	0.01	88.2–104.5	This study
LC-MS/MS	Vegetables	0.002	0.007	85.3–95.7	[41]
HPLC-DAD	Fruits	0.01	0.03	78.2–89.5	[44]
DLLME-GC-MS/MS	Tea	0.008	0.025	80.5–92.1	[42]
Traditional GC-MS	Vegetables	0.01	0.03	75.6–88.3	[43]

**Table 3:** Dinotefuran residue levels in commercial fruits and vegetables.

Name	Concentration (mg/kg)	RSD%	Spiked Concentration (mg/kg)	RSD%
Grape	0.029 ± 0.003	9.01	0.084 ± 0.006	7.43
Nectarine	0.122 ± 0.012	9.56	0.142 ± 0.003	2.38
Watermelon	0.039 ± 0.003	8.63	0.101 ± 0.007	6.89
Cucumber	0.016 ± 0.002	9.48	0.093 ± 0.005	5.52
Spinach	0.077 ± 0.007	9.54	0.209 ± 0.007	3.52
Celery	0.084 ± 0.008	8.96	0.107 ± 0.010	9.34

Note: The spiked concentration is 0.05 mg/kg. The spiked recovery rate is calculated as follows: Spiked Recovery Rate = [(Spiked Concentration − Original Concentration)/Spiked Concentration] × 100%.

**Table 4:** Comparison of dinotefuran residues with national and global monitoring data.

Commodity	This Study	China (2020–2023) *	Global (2020–2023) **	MRL (mg/kg) China/EU/Codex
Grape	0.02–0.03	0.01–0.15	0.01–0.22	0.5/0.1/0.5
Nectarine	0.10–0.13	0.02–0.18	0.01–0.31	0.3/0.1/0.7
Watermelon	0.03–0.04	0.01–0.08	0.01–0.12	0.2/0.1/0.2
Cucumber	0.01–0.02	0.01–0.09	0.01–0.15	0.1/0.1/0.1
Spinach	0.07–0.09	0.01–0.12	0.01–0.18	0.3/0.01/0.3
Celery	0.07–0.09	0.01–0.14	0.01–0.19	0.3/0.01/0.3

Note: * Data from China National Monitoring Program (Global Food Mate, https://www.foodmate.net/ (accessed on 10 September 2025); Pesticide Properties DataBase, https://sitem.herts.ac.uk/aeru/ppdb/ (accessed on 11 September 2025)). ** Compiled from EU, US, and Japan monitoring programs (European Food Safety Authority, https://www.efsa.europa.eu/en (accessed on 13 September 2025); Environmental Protection Agency Ecotoxicology Knowledgebase, https://cfpub.epa.gov/ecotox/index.cfm (accessed on 14 September 2025); and Ministry of Agriculture, Forestry and Fisheries (Japan), https://www.maff.go.jp/e/index.html (accessed on 10 September 2025)).

**Table 5:** Comparison of environmental degradation and mobility characteristics of dinotefuran and other neonicotinoid pesticides.

Pesticide	Photolysis Rate	Hydrolytic Stability	Micro-Biological Degradation	Soil Degradation	Degradation in Plants
Dinotefuran	4–14	~360	20–50	10–30	2–12
Imidacloprid	100–200	~30	40–120	100–200	7–25
Thiamethoxam	3–30	~200	20–75	30–100	4–20
Acetamiprid	30–100	~150	10–50	20–40	2–15

Note: All data represent half-life (DT50) values, measured in days. The above data were retrieved from PPDB (Pesticide Properties DataBase, https://sitem.herts.ac.uk/aeru/ppdb/ (accessed on 20 July 2025)); EFSA (European Food Safety Authority, https://www.efsa.europa.eu/en (accessed on 15 July 2025)); FAO/WHO Pesticide Residues (Food and Agriculture Organization of the United Nations, https://www.fao.org/home/en/ (accessed on 17 July 2025)); and EPA ECOTOX (Environmental Protection Agency Ecotoxicology Knowledgebase, https://cfpub.epa.gov/ecotox/index.cfm (accessed on 21 July 2025)).

**Table 6:** Comparison of environmental mobility characteristics of dinotefuran and other neonicotinoid pesticides.

Pesticide	Koc	Leaching Risk	Water Solubility (mg/L)	Vapor Pressure (mPa)	GUS Index
Dinotefuran	30–50	Higher	39,830	0.0017	4.95
Imidacloprid	200–300	Medium	610	0.0000004	3.76
Thiamethoxam	60–100	Very high	4100	0.00000041	5.18
Acetamiprid	150–200	Medium	590	0.0000043	3.83

Note: Koc is the absorption constant of organic chemicals; GUS index is the groundwater ubiquitous index, GUS > 2.8 indicates leaching potential. The above data were retrieved from PPDB (Pesticide Properties DataBase, https://sitem.herts.ac.uk/aeru/ppdb/ (accessed on 20 July 2025)); EFSA (European Food Safety Authority, https://www.efsa.europa.eu/en (accessed on 23 July 2025)); FAO/WHO Pesticide Residues (Food and Agriculture Organization of the United Nations, https://www.fao.org/home/en/ (accessed on 18 July 2025)); and EPA ECOTOX (Environmental Protection Agency Ecotoxicology Knowledgebase, https://cfpub.epa.gov/ecotox/index.cfm (accessed on 20 July 2025)).

**Table 7:** Comparative analysis of metabolic pathways and toxic metabolites of dinotefuran and other neonicotinoid pesticides.

Pesticide	Main Metabolites	Metabolite Toxicity	Bee	Aquatic Organisms	Earthworm
Dinotefuran	UF, DN	Low toxicity	0.023	0.1–1.0	5.2
Imidacloprid	6-Chloronicotinic acid, Nitroso derivatives	Chronically toxic	0.0039	10–30	10.5
Thiamethoxam	Clothianidin	Highly toxic (more active than the parent)	0.004	0.01–0.1	2.8
Acetamiprid	N-Demethylacetamiprid	Moderately toxic	0.15	10–50	20.0

Note: Bee (Expose LD50, μg/bee); aquatic organisms (Fish LC50, mg/L); earthworm (EC50, mg/kg); UF (1-Methyl-3-(tetrahydro-3-furanylmethyl)urea); and DN (Demethylfuran). The above data were retrieved from EFSA (European Food Safety Authority, https://www.efsa.europa.eu/en (accessed on 10 July 2025)); EPA ECOTOX (Environmental Protection Agency Ecotoxicology Knowledgebase, https://cfpub.epa.gov/ecotox/index.cfm (accessed on 20 July 2025)); and MAFF (Ministry of Agriculture, Forestry and Fisheries (Japan), https://www.maff.go.jp/e/index.html (accessed on 20 July 2025)).

**Table 8:** Acute and chronic risk assessment of dinotefuran in fruits and vegetables.

Name	C_max_ (mg/kg)	ESTI (mg/kg)	aHQ (%)	C_m_ (mg/kg)	EDI (mg/kg)	cHQ (%)
Grape	0.031	0.00018	0.09	0.029	0.00017	0.09
Nectarine	0.133	0.00078	0.39	0.122	0.00071	0.36
Watermelon	0.042	0.00024	0.12	0.039	0.00023	0.11
Cucumber	0.018	0.00015	0.04	0.016	0.00014	0.07
Spinach	0.082	0.00068	0.34	0.077	0.00064	0.32
Celery	0.089	0.00074	0.37	0.084	0.00070	0.35

## Data Availability

The data presented in this study are available on request from the corresponding author.
